# Supplementary material for: Fear, stress, anxiety, depression and insomnia related to COVID-19 among undergraduate nursing students: An international survey
Source: PLoS One. 2023 Oct 5;18(10):e0292470. doi: 10.1371/journal.pone.0292470 (PMC10553289; doi:10.1371/journal.pone.0292470)
Supplement: Attachment — Submitted filename: R1 _ Response to Decision Letter.docx [file pone.0292470.s001.docx]

**Reply to the Editors and Reviewers:**

Dear Editor:

Thank you for reviewing our manuscript.

We have amended the manuscript as the reviewers required, addressing their comments below.

|  | **Comments** | **Responses** |
| --- | --- | --- |
|  |  |  |
|  | **Reviewer #1** |  |
|  | I've enjoyed reading the manuscript. It is well-written and clear.  I have only few minor comments/ suggestions. | Thank you for your comments and feedback |
| 1 | I would made clear from the abstract (and in method section) that background information will be used as independent variables. | Additional sentence was added in abstract.  ‘The independent variables included demographic and academic backgrounds, while fear level, stress, anxiety, depression, and insomnia were the dependent variables.’ |
| 2 | I'm not sure if the regression comprises all the independent variables at once. If yes, please clarify. If no, I would suggest to make regression controlling by country. This would allow to strengthen the generalisability of the findings across countries. | Yes.  A sentence specified this on page 7, line 10.  ‘All variables were selected for entry into the logistic regression models.’ |
| 3 | I would avoid the use of the word "psychological distress" (stress, anxiety, depression) and I would rather use the more general "psychological or psychophysical disease or suffering" given the outcome variables used in the present study. | Modified to be psychological disorders |
| 4 | I would strengthen discussion (with more sentences about comparisons across the countries both within the study and conducted worldwide after two years of pandemic) and study implications sections (mainly for nursing and healthcare context). | Additional sentences were added in the discussion part in page 9 and 10 about the differences between the countries in terms of those symptoms. |
|  |  |  |
|  | **Reviewer #2** |  |
|  | I am grateful for the opportunity to review the manuscript Fear, Stress, Anxiety, Depression and Insomnia Related to COVID-19 among Undergraduate Nursing Students: An International Survey. The objective of this research was to assess levels of fear, stress, anxiety, depression, and insomnia among undergraduate nursing students in five countries two years after the start of the pandemic. The study sample consisted of 918 undergraduate students who answered the questionnaire online. After reading the manuscript, I forward the following considerations: | Thank you for your comments and feedback |
| 1 | The introduction is too generic and the importance of this research is not clear. | Additional sentence and preface? were modified to justify the needed for this research?  ‘However, the mental health status of undergraduate nursing students after two years of the COVID-19 pandemic has yet to be explored. The contribution of this study, therefore, is to ascertain the mental health status of nursing students two years after the WHO first identified COVID-19 as a pandemic. This research sheds light on the often overlooked mental health aspect of undergraduate nursing students. Investigating fear, stress, anxiety, depression, and insomnia, it unravels the psychological toll imposed by the pandemic on this group of learners. The study's significance lies in its potential to inform tailored support strategies and interventions to bolster the mental well-being of nursing students. As educators, policymakers, and healthcare professionals gain deeper insights, they can develop comprehensive plans to mitigate the adverse effects of the pandemic on the mental health of nursing students. Furthermore, this research adds to the growing body of knowledge on mental health during crises, offering valuable lessons for future preparedness and response measures. Overall, the study's outcomes have the potential to foster a more compassionate and resilient educational environment, emphasizing the importance of prioritizing mental health during these challenging times.’ |
| 2 | The objective variables are not addressed in the introduction, allowing the identification of gaps that will be filled. |  |
| 3 | The justification for carrying out the research after two years of the pandemic is insufficient to justify the study. It should be noted that there are already many publications on this subject, and it is not noticeable what differentiates this research from the others. |  |
| 4 | In the methods there is no characterization of the sample. | Additional sentences (highlighted) were added in the method section explain the institutional. |
| 5 | The survey was carried out via the web and had the premise that students knew the English language. However, there is no way to be sure that this occurred and this may be a bias in this study, since 52.5% of the participants were from Saudi Arabia. | All institutions included in the study have English as the main teaching language. |
| 6 | The study used a scale with a Cronbach's alpha value lower than 0.80, which could be a problem for the instrument's internal consistency. | Thank you for your comments and feedback.  Additional sentences were added explain the Cronbach's alpha.  In addition,  According to Nunnally (1994), an alpha of 0.70 or higher is considered acceptable.  Nunnally JC. Psychometric theory. 3rd ed. New York: McGraw-Hill; 1994. |
| 7 | The results are in agreement with the methods. | Thank you for your comments. |
| 8 | The discussion shows that the study is similar to several other studies already published. The authors must indicate advances in relation to what is already known. | Additional sentences were added (highlighted) in the discussion part explain the result section and compression between different countries. |
|  |  |  |
|  | **Reviewer #3**  Please see specific comments for feedback. Thank you for the opportunity to review this interesting manuscript. Overall, this is a well-designed study and well-organized manuscript. It would benefit from copyediting (see several comments regarding this) and further development of some areas. | Thank you for your comments and feedback |
| 1 | Ethics Statement did not address the form of consent obtained | The consent obtaining were addressed on page 4:  The consent statement was presented on the first screen of the survey tool and participants were required to click a button to confirm their consent before they could complete the survey.  And page 11  Informed consent:  Informed consent was obtained from all individual participants included in the study. |
| 2 | Data availability does not seem to meet the requirements of the publication (available only on request, without further explanation of reason for this). | Modified the statement of the data availability as below:  ‘As the data are collected from four countries, they are not publicly available due to the Rules Governing the Ethics of Scientific Research. Researchers may obtain access to the data directly from the principal investigator, mhamedan@hotmail.com. |
| 3 | The manuscript refers to “5 countries” but only four are described: UAE, Saudi Arabia, UK, and Oman. I’m wondering if there might be several countries within the UK that contributed, but this needs further clarification. | Modified the manuscript to be four countries. |
| 4 | p. 3 line 12-13: please rephrase for clarity “many nursing students were needed to undertake clinical practice placements in addition to their education.” Do you mean that healthcare facilities needed the students to complete practice placements or that students needed these placements for their education? | Modified to be.  ‘Nursing students experienced a decrease in traditional classroom learning due to the pandemic, and at the same time, they were required to engage in essential clinical practice places as part of their education. This double impact affected their learning experience and demanded additional responsibilities during their training.’ |
| 5 | p. 3 lines 14-15: delete second “that” in “It is known that, even outside the context of a global pandemic, that nursing students…” | second “that” has been deleted |
| 6 | Additional information about the universities involved would be helpful. Was there just one institution per country? It is implied that all offer a 4-year degree but this is not explicit. It would be helpful to know this information and any other information that might help readers know if these results may be applicable to their settings. | Two sentences were added in method section 2.1.  ‘This international collaborative study used a web-based cross-sectional design and was conducted in four countries (KSA, Oman, UK, and UAE). The sample consisted of undergraduate nursing students who were attending higher education institutions in participating countries (UK - Northumbria University, Oman - Oman College of Health Sciences, Saudi Arabia - Jazan University, UAE - Fatima College of Health Sciences).’ |
| 7 | 2.1 Study Design: Since students participated in this study it would be helpful to know if they were approached by researchers who were or were not directly involved in their education. Along the same lines, was it made explicit to potential participants that participation in the study would not affect their grades/standing in their program? | Thank you for your comments.  As mentioned in the methods section, we utilized a web-based cross-sectional design and sent anonymous surveys to all the participants. The researcher did not have any direct contact with the students. |
| 8 | p. 4 lines 16-17: “being an pre-registration nursing student”: please change “an” to “a” | Changed to be “a” |
| 9 | p. 4 line 23: “Demographic and academic background data were collected included age, sex…” Please change wording to make this sentence clearer. | Changed to be.  “Data on demographic and academic backgrounds were gathered, encompassing information such as” |
| 10 | p. 5 lines 9-10: If potential responses range from 0-4, wouldn’t this be a 5-point scale? | Thank you for the comments. The range will be as following: 0 \| 1 \| 2 \| 3 \| 4; Never \| Almost never \| Sometimes \| Fairly often \| Very often In total will be 5-point scale |
| 11 | p. 5 line 11: formatting of reference should be changed (Jahrami et al., 2020) | Changed and modified |
| 12 | 2.2 Measures: have any of these been used in this population or a similar ones before (students, nurses, nursing students)? | Yes  Additional sentences were added to 2.2 Measures section:  Highlighted |
| 13 | p. 5 line 18: reference format should be changed (Zigmond and Snaith, 1983) | modified |
| 14 | 2.2 Measures: please be more explicit about the cutoff scores adopted for this study for the PSS and ISS. | Sentences were highlighted.  The cutoff:  PSS ≥14  ISI ≥10 |
| 15 | 2.3 Data Analysis: Were data screened for missing, implausible, and outlier values? | Two sentences were added.  ‘The questionnaires were thoroughly reviewed for accuracy and completeness after the data collection process. Participants who decided not to respond to certain questions or left one or more questions unanswered were not included in the valid data set due to missing or incomplete data.’ |
| 16 | 3 Results: what was the response rate for the survey? How much data were missing in the questionnaires received? | Thank you for your comments.  We distributed the questionnaire using a web-based platform; however, we were unable to determine the exact number of respondents to calculate the response rate.  A sentence explaining invalid questionnaire was added.  ‘A total of 1053 questionnaires were collected. After excluding incomplete questionnaires, the study was left with 918 valid questionnaires from nursing students.’ |
| 17 | p. 6 line 13: “A total of 918 nursing students participated on the study.” Please change “on” to “in” | Changed |
| 18 | p. 6 lines 18-20: It would help the reader if you could present the means (SDs) for each instrument immediately next to the name of the instrument. | Modified  ‘The mean (SD) of FCV-19S was 16.86 (SD=6.14), PSS was 21.87 (SD=5.64), HADS-A was 10.25 (SD=3.53), HADS-D 9.11 (SD=3.53), and ISI 12.97 (SD=5.17).’’ |
| 19 | p. 6 lines 20-21 “The prevalence of each construct using the identified cut-off score for each of scale” Please re-word for clarity | Modified  ‘Table 1 shows the prevalence rates of each scale, as follows: fear 46.2%, stress 91.6%, anxiety 69.1%, depression 59.8%, and insomnia 73.2%, based on the specified cut-off scores for each scale.’ |
| 20 | p. 7 line 17: Please change “iof” to “of” | Changed |
| 21 | p. 7 lines 18-19 “Only being a student from Oman appeared to be the most potent predictor of anxiety” Please clarify; was this the only predictor of anxiety or the strongest predictor of anxiety? | Modified  “Only being a student from Oman appeared to be a predictor of anxiety” |
| 22 | p. 7 line 20: please delete “first” | Deleted |
| 23 | p. 7 line 24: please fix spelling of “adamic” | Modified to be “academic” |
| 24 | p. 8 line 9: Mulyadi et al. (2021): please change reference format | Changed |
| 25 | p. 8 lines 11-13: Please clarify if this was during the COVID-19 pandemic: “In addition, prevalence rates in this study were higher in comparison with those in the general  12 population in a meta-analyses of 999 studies where rates were: stress (32%) anxiety (28%),  13 depression (27%), and insomnia (32%) [24].” | Sentence were Modified.  “In addition, prevalence rates in this study were higher in comparison with those in the general population in a meta-analysis of 999 studies where rates were: stress (32%) anxiety (28%), depression (27%), and insomnia (32%) during the COVID-19 pandemic [28]. ” |
| 26 | p. 8 lines 13-14 “The differences between the findings might be explained by the various isolation measures that were used by the countries to limit the spread of COVID-19.” This is one explanation but needs further explanation for those not familiar with isolation measures in the countries studied; were these measures stricter or longer lasting in the countries in this study compared to those included in the reviews you cite? | Thank you for your comments.  Additional explanations were added in next paragraphs. |
| 27 | p. 8 “This is important as the timing of the implementation of these measures can influence the severity of the adverse psychological sequelae.” This statement should be supported by a reference. | Reference was added |
| 28 | p. 8 “This may be explained by those studies having been conducted at the start of the pandemic when there was greater uncertainty of the effects of COVID-19 on humans.” Could this also be explained by the availability of a vaccine at the time of your study compared to vaccine availability in the studies in Thailand and Mexico? | Additional sentence was added  ‘Another possible reason might also be attributed to differences in vaccine availability and distribution, impacting individuals' perception of safety and well-being between the studies.’ |
| 29 | p. 8-9 “The reasons for this might be due to variation in individual countries at the time of conducting the study in terms of the level and nature of COVID-19 restrictions including social isolation, lockdown, and the associated economic challenges.” You may not have an answer to this, but if you do, it may be helpful for your readers to know what sort of country to country differences could explain the significant differences found between countries. | Two sentences were added.  ‘Additionally, the variations in mental health outcomes among countries can also be influenced by factors like the availability and accessibility of healthcare services, public health infrastructure, cultural attitudes towards mental health, and the level of social support systems in place during the pandemic. These country-specific elements contribute to the diverse impacts on individuals' mental well-being during the global health crisis.’ |
| 30 | Several times the authors mention data about how many participants were assigned to clinical placement during the pandemic, but it isn’t clear that this data was collected in section 2.2 | Additional sentence was added in section 2.2  ‘Additionally, students were asked about their COVID-19 infection, vaccination status, and participation in clinical placements throughout the pandemic.’ |
| 31 | p. 9 line 14: “Academic year of study was the strongest predictor of depression, namely second year) and…” please revise for clarity; possible remove the parenthesis. | Modified |
| 32 | p. 9 lines 19-20: “Thus, in future pandemics there may be a need to prioritise supportive interventions for these students.” Do you think these finding might apply to other situations besides pandemics? | Yes, can be applied.  Additional two sentences were added.  ‘Thus, in future pandemics there may be a need to prioritise supportive interventions for students during pandemics can potentially apply to other situations besides pandemics. The underlying principle is that during any crisis or challenging circumstances, individuals may face increased stress, anxiety, depression , insomnia and disruptions in their lives. This can be particularly true for students who might be dealing with additional pressures related to their education and personal lives, such as financial burdens, family responsibilities, health concerns, or social challenges. ’ |
| 33 | p. 9 line 24: please change “will” to “well” | Changed |
| 34 | p. 10 lines 1-2: “Unfortunately, nursing programs not provide sufficient training in how to cope with crises” please revise for clarity | Modified  “Regretfully, research has shown that nursing programs generally do not offer sufficient training on crisis-coping strategies” |
| 35 | p. 10 lines 5-6: “The participants were recruited via online platform and email, which may have reduced limited the response rate…” please revise for clarity | Modified  “Recruitment of participants through online platforms and emails might have led to a lower response rate as some individuals may not regularly check and respond to their emails.” |
| 36 | p. 11 lines 2-3: “training students how to take care of the infection diseases patient” Please consider using “person first” language (for example, “training students how to take care of a patient with an infectious disease”). | Modified |
| 37 | Table 1: The abbreviation KSA has not been used elsewhere in the manuscript and needs explanation please | Kingdom of Saudi Arabia (KSA) Abbreviation were given in the manuscript and highlights |
| 38 | Table 2: In the manuscript it states that “For all analyses performed, a P < .05 was considered statistically significant.” However, this table has flagged results with p <.01. | Thank you for your comments.  Yes, this as the result of the Pearson’s correlation coefficient which given as p <.01. |
