# Supplementary material for: Fear, stress, anxiety, depression and insomnia related to COVID-19 among undergraduate nursing students: An international survey
Source: PLoS One. 2023 Oct 5;18(10):e0292470. doi: 10.1371/journal.pone.0292470 (PMC10553289; doi:10.1371/journal.pone.0292470)
Supplement: Attachment — Submitted filename: Response to Decision Letter 15-9-2023.docx [file pone.0292470.s002.docx]

**Reply to the Editors and Reviewers:**

Dear Editor:

Thank you for reviewing our manuscript.

We have amended the manuscript as the reviewers required, addressing their comments below.

|  | **Comments** | **Responses** |
| --- | --- | --- |
|  |  |  |
|  | **Journal Requirements:** |  |
| 1 | Please review your reference list to ensure that it is complete and correct. If you have cited papers that have been retracted, please include the rationale for doing so in the manuscript text or remove these references and replace them with relevant current references. Any changes to the reference list should be mentioned in the rebuttal letter that accompanies your revised manuscript. If you need to cite a retracted article, indicate the article’s retracted status in the References list and also include a citation and full reference for the retraction notice. | Double checked |
|  |  |  |
|  | **Reviewer #3** |  |
|  | Thank you for your careful attention to the comments provided by the reviewers. I believe that this manuscript has been strengthened by these revisions. I have one minor points that I believe the authors should still address: the PSS is still described as a 4-point scale in the revised manuscript, but the authors stated in their response to reviewers table that it was a 5-point scale (p. 6 lines 4-5). | Thank you for the comments. Yes, the reviewer is correct, and we have modified the sentence on the text. The range will be as following: 0 \| 1 \| 2 \| 3 \| 4; Never \| Almost never \| Sometimes \| Fairly often \| Very often In total will be 5-point scale The sentences in the manuscript page 6 line 4-5 was modified. ‘Each is rated on a 5-point response scale (from 0 to 4).’ |
